# 

**Published:** 1884-03

**Authors:** 


					﻿MARCH, 1884.
THIRTY-FIRST YEAR.
HAITJS
J0UB.XAL
OF
IQnCS A T nPTOT
jd.fia.aju IM
Guaranteed Circulation 200,000 Copies in 12 Months.
E. H. GIBBS, A.M., M.D., Editor, ~
No. 21 CLINTON PLACE, EIGHTH STBEET NET TOBE,
TERMS: $1 a Year.
Siegle number, 10 cts.
				

